# Identification of human host factors required for beta-defensin-2 expression in intestinal epithelial cells upon a bacterial challenge

**DOI:** 10.1038/s41598-024-66568-y

**Published:** 2024-07-04

**Authors:** Weronika Wozniak, Emmanuel Sechet, Yong-Jun Kwon, Nathalie Aulner, Lionel Navarro, Brice Sperandio

**Affiliations:** 1 Institut de Biologie de l’École Normale Supérieure (IBENS), Centre National de la Recherche Scientifique (CNRS) UMR8197, Institut National de la Santé et de la Recherche Médicale (INSERM) U1024, Université PSL, Paris, France; 2 Institut Pasteur, Université Paris Cité, Paris, France; 3 Institut Pasteur Korea, Seoul, South Korea; 4 Present Address: Luxembourg Institute of Health, Dudelange, Luxembourg

**Keywords:** Innate immunity, Human intestinal epithelial cell, Antimicrobial peptide, Beta-defensin-2, Gene regulation, Bacterial challenge, Antimicrobial responses, Innate immunity, Mucosal immunology

## Abstract

The human intestinal tract is colonized with microorganisms, which present a diverse array of immunological challenges. A number of antimicrobial mechanisms have evolved to cope with these challenges. A key defense mechanism is the expression of inducible antimicrobial peptides (AMPs), such as beta-defensins, which rapidly inactivate microorganisms. We currently have a limited knowledge of mechanisms regulating the inducible expression of AMP genes, especially factors from the host required in these regulatory mechanisms. To identify the host factors required for expression of the beta-defensin-2 gene (*HBD2*) in intestinal epithelial cells upon a bacterial challenge, we performed a RNAi screen using a siRNA library spanning the whole human genome. The screening was performed in duplicate to select the strongest 79 and 110 hit genes whose silencing promoted or inhibited *HBD2* expression, respectively. A set of 57 hits selected among the two groups of genes was subjected to a counter-screening and a subset was subsequently validated for its impact onto *HBD2* expression. Among the 57 confirmed hits, we brought out the TLR5-MYD88 signaling pathway, but above all new signaling proteins, epigenetic regulators and transcription factors so far unrevealed in the *HBD2* regulatory circuits, like the GATA6 transcription factor involved in inflammatory bowel diseases. This study represents a significant step toward unveiling the key molecular requirements to promote AMP expression in human intestinal epithelial cells, and revealing new potential targets for the development of an innovative therapeutic strategy aiming at stimulating the host AMP expression, at the era of antimicrobial resistance.

## Introduction

The efficacy of antimicrobial defense in the human intestinal tract relies on the ability of the mucosal immune system to recognize and neutralize microorganisms. In this context of innate immunity, sensing of invading bacteria occurs mainly through engagement of host cell Toll-like receptors (TLRs) and other pattern-recognition molecules, such as the nucleotide oligomerization domain (NOD) proteins, by microbe-associated molecular patterns (MAMPs)^1,2^. After the microorganism recognition step, the subsequent antimicrobial response is achieved by recruitment of immune cells and synthesis of antimicrobial factors by the mucosa, including production of antimicrobial peptides (AMPs) such as cathelicidins and defensins that exhibit a broad spectrum of activities against a wide range of bacteria.

Among AMPs, defensins represent a family of cationic peptides with a common beta-sheet core stabilized with three disulfide bridges between six conserved cysteine residues, and are subdivided into alpha-, beta- and theta-defensins on the basis of the linkage of cysteine residues. Among them, the beta-defensins are ubiquitous and present in all vertebrates. The human genome has more than 30 genes coding for beta-defensins, which are produced mainly by epithelial cells^3,4^. In intestinal epithelial cells, expression of the main beta-defensin genes, *HBD1-4*, is either constitutive or inducible in response to various stimuli. Expression of *HBD1* is essentially constitutive, whereas expression of *HBD2-4* is inducible in response to several signals, including proinflammatory cytokines and MAMPs^3^.

Beta-defensins have a broad spectrum of antimicrobial efficacy and act on Gram-positive and Gram-negative bacteria as well as on fungi, parasites and enveloped viruses^5^. They exert their action by mainly interacting with the surface of microorganisms. They bind the anionic portion of the lipopolysaccharide (LPS) molecule in Gram-negative bacteria, while they interact with teichoic acids or with anionic groups present in the peptidoglycan molecule in Gram-positive bacteria. The interaction of beta-defensins with different structures of the bacterial cell envelope permeabilizes the membrane either through a detergent effect resulting in the leakage of cytoplasmic components, or through the formation of pores subsequent to peptide aggregation. This phenomenon coincides with inhibition of RNA, DNA and protein synthesis, and with a decrease of the bacterial viability^6^.

Currently, the growing threat of antimicrobial resistance in bacteria and the need for new molecules with antibiotic activities are stimulating interest in the use of beta-defensins as candidate therapeutic molecules. The direct antimicrobial activities of these AMPs, coupled to their additional protective properties such as modulation of the immune response, wound healing, angiogenesis, tissue remodeling, and ability to bind LPS in septic shock models, make them therapeutic targets with high valuable potential^7,8^. Facing this, clinical studies and recent progress in molecular medicine show that several human diseases, especially inflammatory bowel diseases (IBDs), are associated with defects in the expression of beta-defensins^9,10^. These advances in the comprehension of the role played by AMPs at the host-microbe interface and their potential use to maintain or promote intestinal homeostasis are however hampered by a poor knowledge of mechanisms regulating their gene expression.

Here, we used a model of human intestinal epithelial cells challenged by a bacterium, and a genome-wide RNAi screening approach, to identify the host factors required to regulate expression of the *HBD2* gene (also known as *DEFB4A*) encoding the human beta-defensin-2. A primary screening allowed the identification of 79 and 110 genes encoding proteins promoting or inhibiting *HBD2* expression, respectively. A subsequent counter-screening on a subset of 57 genes, followed by targeted confirmations, allowed to validate 95% of them, notably encoding cell signaling proteins (e.g. MYD88), chromatin regulators (e.g. SUDS3), or transcription factors (e.g. ZNF3, GATA6). This insight into the expansive and multi-layered landscape of *HBD2* transcriptional regulation is crucial for unraveling its fundamental role in the host defense and high valuable for developing an innovative therapeutic strategy aiming at stimulating its endogenous expression at mucosal surfaces.

## Results

### Selecting a bacterium inducing *HBD2* gene expression

To identify factors from the host that are required for the regulation of the expression of the beta-defensin-2 gene (*HBD2*) in human intestinal epithelial cells, we started by looking for a bacterium that induces *HBD2* without invading cells, as can do a pathogen like *Shigella* or *Listeria*^11^. To this end, we investigated bacterial species like the *Escherichia coli* K12 and *Streptococcus macedonicus* commensals, and the *E. coli* LF82 and *S. gallolyticus* pathobionts. For this purpose, we established an in vitro model based on monolayers of confluent human intestinal epithelial TC7 cells challenged with bacteria, by testing multiplicities of infection (MOI) ranging from 1 to 100 bacteria per cell. This model had the double advantage to mimic the epithelial lining and to allow the detection of transcripts of most of human intestinal antimicrobial peptide genes. Bacterial challenges were performed on cell monolayers from 1 to 6 h, and cell RNA was harvested and analyzed by qPCR for the expression of *HBD2.*

**Figure 1:**
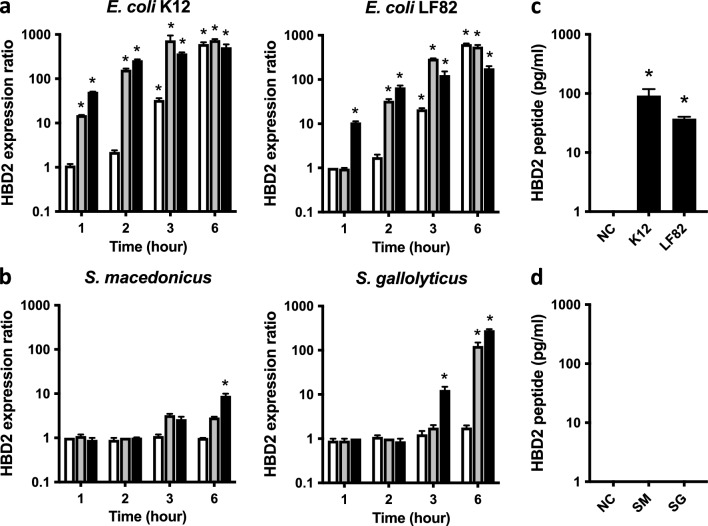
Expression of *HBD2* in TC7 cells challenged with *Escherichia coli* or Streptococci strains. (**a**) Transcription of *HBD2* in wild-type TC7 cells challenged for 1–6 h with the *Escherichia coli* K12 (commensal) or *E. coli* LF82 (pathobiont) Gram-negative bacteria, and (**b**) the *Streptococcus macedonicus* (commensal) or *Streptococcus gallolyticus* (pathobiont) Gram-positive bacteria. Values are presented on a logarithmic scale as the ratio of gene transcription in challenged cells compared with non-challenged cells, using a MOI = 1 (white bars), MOI = 10 (gray bars) or MOI = 100 (black bars). Data are presented as the mean ± s.d. (n = 3 biological replicates). *P < 0.05 evaluated by two-tailed Mann–Whitney U test. (**c**) ELISA dosage of the HBD2 peptide in supernatants of cells challenged for 3 h with *E. coli* K12 or *E. coli* LF82, and (**d**) *S. macedonicus* or *S. gallolyticus*, using a MOI of 10 bacteria per cell. Bacterial challenges were stopped by an antibiotic treatment and supernatants were collected 24 h after the beginning of challenges. Values are presented on a logarithmic scale in picogram of peptide per milliliter. NC: non-challenged cells; K12: cells challenged with *E. coli* K12; LF82: cells challenged with *E. coli* LF82; SM: cells challenged with *S. macedonicus*; SG: cells challenged with *S. gallolyticus*. Data are presented as the mean ± s.d. (n = 3 biological replicates). *P < 0.05 evaluated by two-tailed Mann–Whitney U test.

In nonchallenged cells, we observed a very low basal level of *HBD2* expression, relating to the inducible nature of the gene. Challenge of cells with *E. coli* K12 or *E. coli* LF82 was followed by a MOI-dependent induction of *HBD2* transcription over its basal level, observed as soon as 1 h after contact of cells with bacteria (Fig. 1a). The strongest *HBD2* induction was observed with *E. coli* K12 at 3 h post-challenge (740-fold), using a MOI of 10 bacteria per cell. Conversely, challenge of cells with *S. macedonicus* or *S. gallolyticus* showed a much lower induction of *HBD2* expression, regardless of the MOI or timepoint tested (Fig. 1b). The strongest induction of *HBD2* was detected with *S. gallolyticus* at 6 h post-challenge, using a MOI 100 (280-fold). Collectively, these results indicate that Gram-negative *E. coli* induce more *HBD2* expression compare to Gram-positive Streptococci. Among them, the *E. coli* K12 commensal is the strain inducing the fastest and highest *HBD2* transcription, in our experimental setting.

To further characterize induction of the *HBD2* gene expression in cells upon bacterial challenges, we next investigated production of the HBD2 peptide. For this purpose, cells were challenged for 3 h with *E. coli* K12, *E. coli* LF82, *S. macedonicus* or *S. gallolyticus*, using a MOI of 10 bacteria per cell. Then, bacteria were killed by an antibiotic treatment, and ELISA dosages were performed on cell supernatants collected 24 h after the beginning of each challenge to quantify the HBD2 peptide secretion. HBD2 was not detected in supernatants of non-challenged cells (Fig. 1c, d). In contrast, supernatants of cells challenged with *E. coli* revealed increased concentrations of the HBD2 peptide, in a strain-dependent manner (Fig. 1c). Quantitatively, concentration of HBD2 was measured at 92 and 37 pg/mL upon challenge with *E. coli* strains K12 and LF82, respectively. Conversely, the peptide was not detected in supernatants of cells challenged for 3 h with *S. macedonicus* or *S. gallolyticus* (Fig. 1d). Together, these data show that cell challenges induce transcription of the *HBD2* gene, as well as production of the HBD2 peptide, in a bacterium-dependent manner.

### Developing a stable reporter cell line to monitor the inducible expression of *HBD2*

To investigate host factors that are involved in regulation of *HBD2* expression upon a bacterial challenge, we developed a stable human intestinal reporter cell line allowing an expression monitoring of this gene by microscopy. The construction was developed in the human intestinal epithelial TC7 cell line, in which cells harbor a transcriptional fusion between the endogenous *HBD2* promoter and the enhanced GFP reporter gene. Because a CRISPR-Cas9 genome editing approach was not successful in the TC7 cell line, the construction was delivered and stably integrated in its genome by a lentiviral vector. Several clones were selected using an antibiotic marker and characterized based on *GFP* expression driven by the ectopic *HBD2* promoter, in comparison to *HBD2* gene expression at its genomic locus (Supplementary Fig. S1).

**Figure 2:**
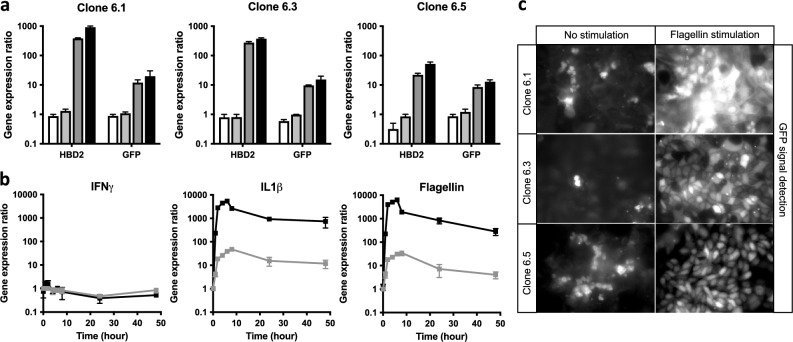
Characterization of stable TC7 reporter clones to monitor *HBD2* expression. (**a**) Transcription of *HBD2* and *GFP* genes in clones 6.1, 6.3 and 6.5 treated for 24 h with 20 ng/mL gamma-interferon (white bars), 20 ng/mL interleukin-17 (light gray bars), 20 ng/mL interleukin-1-beta (dark gray bars), or 5 μg/mL flagellin (black bars). Values are presented on a logarithmic scale as the ratio of gene transcription in treated cells compared with non-treated cells. Data are presented as the mean ± s.d. (n = 4 biological replicates). (**b**) Transcriptional kinetic of *HBD2* (black line) and *GFP* (gray line) genes in cells treated with 20 ng/mL gamma-interferon, 20 ng/mL interleukin-1-beta, or 5 μg/mL flagellin. Values are presented on a logarithmic scale as the ratio of gene transcription in treated cells compared with non-treated cells. Data are presented as the mean ± s.d. (n = 4 biological replicates). (**c**) GFP signal detection in clones 6.1, 6.3 and 6.5 treated for 24 h with 5 μg/mL flagellin, compared with non-treated clones. Experiments were performed on coverslips in 12-well plates (0.75 × 10^6^ cells/well). Signal was detected by epifluorescence microscopy at 40 × magnification. Images are representative of 3 biological replicates.

Three independently isolated clones (i.e. 6.1, 6.3, 6.5) were stimulated by different molecules described in the literature to induce *HBD2* expression in human cells or not at all (Fig. 2). These molecules are gamma-interferon (IFNγ), interleukin-17 (IL17), interleukin-1-beta (IL1β), and the flagellin MAMP. Cell monolayers from each clone were grown and stimulated by the molecules for 24 h. Cell RNA was harvested and then analyzed by qPCR for (i) expression of the *HBD2* gene at its locus, and (ii) expression of the stably integrated *GFP* reporter gene driven by the *HBD2* promoter. In non-stimulated cells, we observed a basal level of expression for *HBD2* and *GFP* genes. Stimulation of clones with IL1β or flagellin was followed by a transcriptional induction of both *HBD2* and *GFP* genes, over their basal level of expression (Fig. 2a). The level of induction was however higher for *HBD2* than for *GFP*. Regarding clone 6.1, which is the most responsive of the three clones to the stimulations, *HBD2* was induced 380-fold and 920-fold by IL1β and flagellin, respectively, compared to 12-fold and 20-fold for the *GFP* gene. In contrast, stimulation of clones with IFNγ or IL17 was not followed by any induction of *HBD2* nor *GFP* expression in the three studied clones. Together, these data show that the *GFP* reporter gene responds to an IL1β and flagellin stimulation, as observed for the *HBD2* gene. The level of *GFP* induction is lower than *HBD2*, but the expression pattern of the two genes is similar. Clone 6.1 is the one, among those studied, showing the highest level of induction for both genes.

We then investigated the kinetic of *HBD2* and *GFP* gene expression in clone 6.1 (Fig. 2b). Experiments were performed on cell monolayers stimulated with IFNγ, IL1β or flagellin. Following treatment, RNA was extracted at several timepoints and analyzed by qPCR. Under these experimental conditions, we detected a transcriptional induction of *HBD2* and *GFP* as early as 1 h after stimulation of cells with IL1β or flagellin. Expression of both genes showed a maximum of induction at 6 h post-treatment, followed by a gradual decrease measured up to 48 h. Quantitatively, flagellin-mediated induction ratios of *HBD2* and *GFP* genes were 6300-fold and 35-fold at the peak, respectively, and 280-fold and fivefold at 48 h post-stimulation. As control, expression of *HBD2* and *GFP* was found not induced in cells treated with IFNγ, at any time. Although the induction level of *GFP* was found lowest than the *HBD2* one, these results indicate collectively that the kinetic of expression of both genes is comparable and follow a similar pattern in response to a same stimulus.

To confirm that the *GFP* gene driven by the *HBD2* promoter is indeed translated in a functional reporter protein, we finally analyzed the GFP signal produced by the three clones independently selected (Fig. 2c). To this end, we performed a detection of the GFP signal in monolayer of cells stimulated for 24 h with flagellin, by epifluorescence microscopy. In non-stimulated cells, we observed a basal GFP signal regardless of the clone analyzed. Detection of this basal level of GFP signal was directly related to the basal level of *GFP* transcription previously observed. Conversely, stimulation of clones with flagellin was followed by a strong GFP signal detected above respective basal levels. Intensity of the GFP signal was higher in clone 6.1 than in the others, in agreement with the transcriptional analysis (Fig. 2a, c). Together, these data confirm that transcriptional induction of the *GFP* gene is followed by translation of the GFP reporter. Intensity of the GFP signal detected in cells is proportionally correlated with the level of *GFP* transcription. Clone 6.1 is the one among those characterized showing the highest GFP induction at the transcriptional and translational levels, upon stimulation by an inducer molecule.

### Screening a genome wide siRNA library to identify host factors promoting or inhibiting *HBD2* expression upon an *E. coli* challenge

To identify factors from the host promoting or inhibiting transcription of the *HBD2* gene upon a bacterial challenge, we performed a siRNA genome-wide screening using the *HBD2* reporter cell line (i.e. clone 6.1) challenged with *E. coli* K12. The screening was performed in duplicate, in two independent experiments, using a siRNA library targeting 18,236 human genes. After transfection, cells were incubated for 48 h to allow gene silencing and challenged for 3 h with *E. coli* at a MOI of 10. Cells were then washed, incubated in fresh complete media with antibiotics for additional 24 h, before their fixation and analysis of the GFP signal (Supplementary Fig. S2).

**Figure 3:**
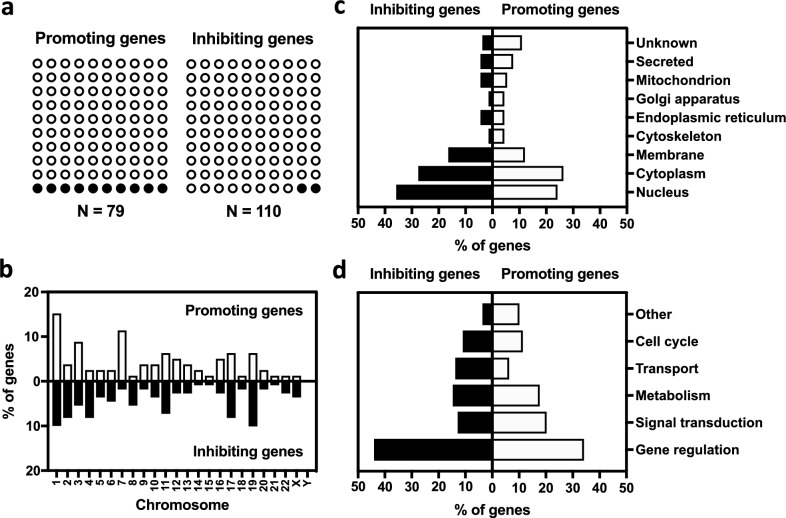
Functional analysis of genes promoting or inhibiting *HBD2* expression. (**a**) Occurrence of *HBD2* with promoting or inhibiting genes in the literature. The association queries between “*HBD2”* and the “hit gene” were submitted to PubMed (NCBI). Results are presented as the percentage of matches linking “*HBD2*” with the “hit gene” (black dots), compared to no matches (white dots), for promoting and inhibiting genes. (**b**) Genomic distribution of genes promoting or inhibiting *HBD2* expression. Promoting (white bars) and inhibiting (black bars) genes were mapped in the human genome using GenBank (NCBI). Results are presented as the percentage of promoting or inhibiting genes located on each of the 23 chromosome pairs. (**c**) Subcellular locations of proteins encoded by genes promoting or inhibiting *HBD2* expression. Promoting (white bars) and inhibiting (black bars) proteins encoded by the identified genes were classified using UniProtKB (UniProt). Results are presented as the percentage of promoting or inhibiting genes distributed according to their cellular location among nucleus, cytoplasm, membrane, cytoskeleton, endoplasmic reticulum, Golgi apparatus, mitochondrion, secreted, or unknown. (**d**) Biological processes involving proteins encoded by genes promoting or inhibiting *HBD2* expression. Promoting (white bars) and inhibiting (black bars) proteins encoded by the identified genes were categorized using UniProtKD (UniProt). Results are presented as the percentage of promoting or inhibiting genes distributed according to their involvement in biological processes among gene regulation, signal transduction, metabolism, transport, cell cycle, or other.

We identified 79 and 110 host genes promoting or inhibiting expression of *HBD2*, respectively (Supplementary Table S1, Supplementary Table S2). An extensive bibliographic study revealed that only 10% of promoting genes, and 2% of inhibiting genes, were previously known to be directly or indirectly linked to the *HBD2* gene expression or its encoded peptide (Fig. 3a). This is the case of *TLR5* and *MYD88* promoting genes which were already known to be involved in the signaling pathway leading to *HBD2* transcription^12,13^. An analysis of the genomic location of genes promoting or inhibiting *HBD2* expression revealed that they were distributed on all pairs of human chromosomes, with the exception of the Y chromosome (Fig. 3b). As an example, among the 79 genes promoting *HBD2* expression, 15% of them are located on the first pair of chromosomes. We then analyzed the subcellular location of proteins encoded by genes promoting or inhibiting *HBD2* expression (Fig. 3c). To this end, we used the public-free UniProtKB database collecting functional information on proteins. We classified these proteins according to 9 cellular locations among nucleus, cytoplasm, membrane, cytoskeleton, endoplasmic reticulum, Golgi apparatus, mitochondrion, secreted and unknown. As such, we found that 25% and 35% of proteins involved in the promotion or inhibition of *HBD2* expression were annotated as nuclear proteins, respectively. We finally investigated the biological processes in which proteins encoded by genes promoting or inhibiting *HBD2* expression are involved (Fig. 3d). Using the same database, we divided proteins into 6 processes, including gene regulation, signal transduction, metabolism, transport, cell cycle and other. As an example, following this categorization process, we found that 34% and 44% of proteins promoting or inhibiting *HBD2* expression were annotated as gene regulating proteins, respectively. Collectively, these data show that most of genes identified as promoting or inhibiting *HBD2* expression predominantly encode signaling proteins, as well as epigenetic regulators and transcription factors. As a control, bringing out the TLR5 and MYD88 regulatory proteins previously identified as part of the *HBD2* regulatory circuits, validates our experimental approach.

### Validating genes promoting or inhibiting *HBD2* expression by counter-screening and targeted confirmation

**Figure 4:**
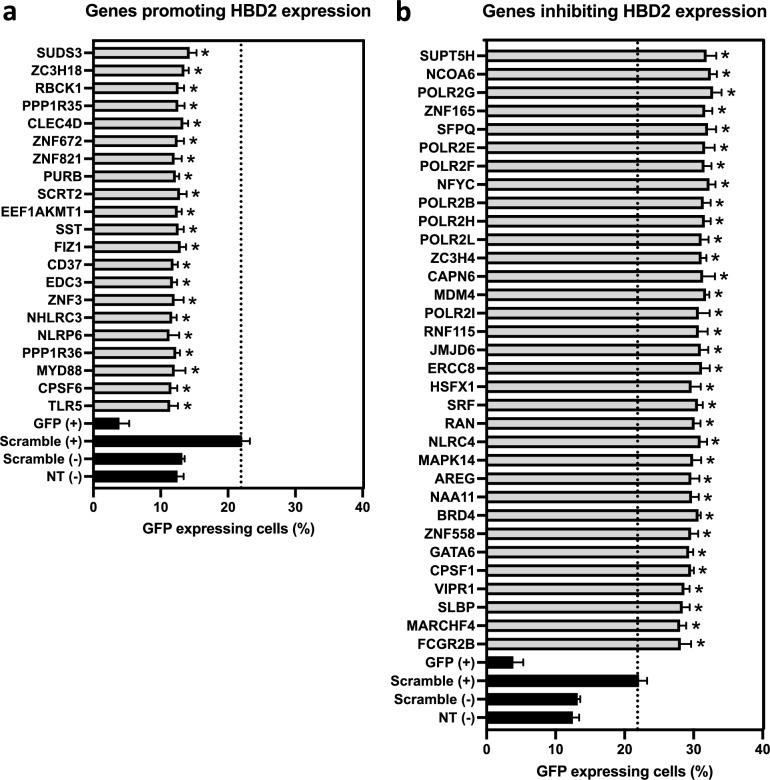
Validation of a set of genes promoting or inhibiting *HBD2* expression by counter-screening. A subset of genes selected among those identified in the primary screening, as (**a**) promoting or (**b**) inhibiting *HBD2* expression, was subjected to a validation step by re-analyzing their impact on *HBD2* transcription in 4 independent siRNA transfection experiments. Results are presented as the percentage of GFP expressing cells, as the mean ± s.d. (n = 4 biological replicates). Grey bars: target siRNA; black bars: control siRNA. GFP (+): cells transfected with GFP siRNA and challenged with *E. coli*; scramble (+): cells transfected with scramble siRNA and challenged with *E. coli*; scramble (−): cells transfected with scramble siRNA and non-challenged; NT (−): non-transfected and non-challenged cells. *P < 0.05 evaluated by One-way Anova (target siRNA *vs* scramble (+)).

To validate the host genes identified by the primary screenings as promoting or inhibiting *HBD2* expression upon an *E. coli* K12 challenge, we randomly selected 57 genes (i.e. 22 promoting- and 35 inhibiting-genes) and retested them in a counter-screening assay, in 96-well plates that is a robust and well-established format used for screenings. Four independent experiments were performed on the *HBD2* reporter cell line (i.e. clone 6.1) in presence of *E. coli*, at a MOI of 10 bacteria per cell. Two types of siRNA control were used (i.e. *GFP* and scramble), and each siRNA (i.e. control and target) was tested in four wells per experiment (Supplementary Fig. S3, Fig. 4).

Regarding controls, we observed a basal level of GFP expressing cells reaching 12% in non-transfected and nonchallenged cells (Fig. 4a, b). A similar level of GFP expressing cells was measured in nonchallenged cells transfected with scramble siRNA. Conversely, challenge of scramble siRNA-transfected cells with *E. coli* was followed by an increased level of GFP expressing cells (i.e. 22%.). This value was considered as the reference threshold for data analysis. Finally, as expected for a positive control, challenge of GFP siRNA-transfected cells with *E. coli* led to a decreased level of GFP expressing cells (i.e. 3%). Regarding target genes, transfection of cells with siRNAs against the 22 genes promoting *HBD2* expression was followed by a decreased level of GFP expressing cells upon *E. coli* challenge, compared to the reference threshold (Fig. 4a). Values dropped from 22% to less than 13%, with exception of the *EDC3* gene whose data were not found substantial (Fig. 4a, Supplementary Fig. S4). Conversely, transfection of cells with siRNA targeting the 35 genes inhibiting *HBD2* expression led to an increased level of GFP expressing cells upon the bacterial challenge, compared to the reference threshold (Fig. 4b). Values jumped from 22% to more than 28%, with exception of the *JUP* and *RBM22* genes whose data were not found substantial (Fig. 4b, Supplementary Fig. S4). Together, results of the counter-screening validate 95% of the genes promoting (i.e. 21 genes out of 22) or inhibiting (i.e. 33 genes out of 35) *HBD2* expression upon a bacterial challenge, which were identified in the primary screening.

**Figure 5:**
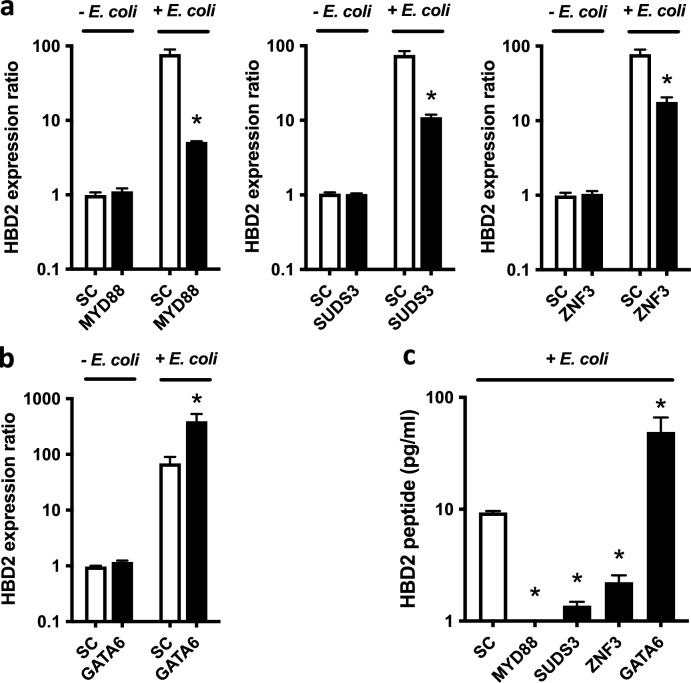
Targeted validation of a set of host factors promoting or inhibiting *HBD2* expression. A set of four host factors selected among those validated by counter-screening, as promoting or inhibiting *HBD2* expression, was subjected to further validation by investigating their impact on *HBD2* transcription in wild-type TC7 cells challenged with *E. coli*. Wild-type cells were silenced for 48 h using siRNA against (**a**) the *MYD88*, *SUDS3* and *ZNF3* promoting genes, or (**b**) the *GATA6* inhibiting gene, and then challenged for 3 h with *E. coli* K12, at a MOI of 10 bacteria per cell. *HBD2* transcription was finally measured by qRT-PCR. Values are presented on a logarithmic scale as the ratio of gene transcription in challenged cells compared with non-challenged cells, following scrambled siRNA (SC, white bars) or target siRNA (black bars) transfection. Data are presented as the mean ± s.d. (n = 3 biological replicates). *P < 0.05 evaluated by two-tailed Mann–Whitney U test. (**c**) ELISA dosage of the HBD2 peptide in supernatants of cells silenced for 48 h using scrambled siRNA (SC), siRNA against the *MYD88*, *SUDS3* and *ZNF3* promoting genes, or siRNA against the *GATA6* inhibiting gene, and then challenged for 3 h with *E. coli* K12, at a MOI of 10 bacteria per cell. The bacterial challenge was stopped by an antibiotic treatment and supernatants were collected 24 h after the beginning of the challenge. Values are presented on a logarithmic scale in picogram of peptide per milliliter. Data are presented as the mean ± s.d. (n = 3 biological replicates). *P < 0.05 evaluated by two-tailed Mann–Whitney U test.

We finally proceeded to targeted confirmations by evaluating the *HBD2* mRNA and HBD2 peptide levels after transfection for 48 h of wild-type TC7 cells with control and target siRNAs, upon challenge with *E. coli* K12 at a MOI of 10. We targeted three *HBD2* promoting genes encoding a signal transduction protein (i.e. *MYD88*), a chromatin regulator (i.e. *SUDS3*) and a transcription factor (i.e. *ZNF3*), respectively, and one *HBD2* inhibiting gene encoding a transcription factor (i.e. *GATA6*). Silencing efficiency reached at least 80% for each of them. Bacterial challenge was performed on cell monolayers for 3 h and cell RNA was harvested and then analyzed by qPCR for expression of *HBD2* (Fig. 5a, b). Quantification of the HBD2 peptide was performed by ELISA assays on supernatants coming from cells challenged for 3 h with bacteria, treated with antibiotics, and collected 24 h after the beginning of the challenge (Fig. 5c).

In non-challenged cells, transcription of *HBD2* was not induced and found similar in all tested conditions. The basal level of *HBD2* transcription was very low, almost undetectable, no variation in its expression was therefore measured upon scramble or target siRNA transfection (Fig. 5a, b). In contrast, in cells transfected with scramble siRNA and challenged with *E. coli*, transcription of *HBD2* was induced 80-fold by the bacterium, compared with non-challenged cells transfected with scramble siRNA (Fig. 5a, b). Concentration of the HBD2 peptide was closed to 10 pg/mL (Fig. 5c). Interestingly, in cells transfected with siRNA targeting *HBD2* promoting genes and challenged with *E. coli*, transcription of *HBD2* was much less induced, with ratios ranging from fivefold upon *MYD88* silencing, to 11- and 20-fold upon *SUDS3* and *ZNF3* silencing, respectively (Fig. 5a). The HBD2 peptide was not detected upon *MYD88* silencing, whereas its concentration was measured at 1.3 and 2.3 pg/mL upon *SUDS3* and *ZNF3* silencing, respectively (Fig. 5c). Conversely, in cells transfected with siRNA targeting the *GATA6* inhibiting gene and challenged with the bacterium, transcription of *HBD2* was much more induced, with a ratio reaching 390-fold (Fig. 5b). Concentration of the HBD2 peptide reached 49 pg/mL (Fig. 5c). Collectively, these data confirm the involvement of these four host factors identified by genome wide screening, in the promotion or inhibition of *HBD2* expression upon a bacterial challenge, in human wild-type intestinal epithelial cells.

## Discussion

The human intestinal epithelium achieves a barrier function between the host and the luminal environment. This barrier protects against invasion and systemic dissemination of both commensal and pathogenic bacteria. Accessibility to the apical surface of epithelial cells and rupture of the physical barrier are among the main properties that distinguish bacterial species. Among the numerous effectors participating in establishment and maintenance of the epithelial homeostasis, antimicrobial peptides that are ubiquitously expressed by epithelial cells and secreted into the mucus layer, throughout the gastrointestinal tract, play a crucial role in controlling both the resident and transient bacterial populations^14^. These defense effectors participate in the innate immune response to commensals under steady-state conditions, and against enteric pathogens when homeostasis of the epithelial barrier is subverted by their crosstalk with mucosal tissues.

Among antimicrobial peptides, the intricate regulations of *HBD2* highlights the complexity of the immune response to these microbial challenges^15,16^. One pivotal signaling pathway involved in *HBD2* expression is the NF-κB pathway. Activation of NF-κB, triggered by Toll-like receptors (i.e. TLR2, TLR4, TLR5) and Nod-like receptors (i.e. NOD1, NOD2), leads to its translocation into the nucleus, where it binds to the *HBD2* promoter and promotes transcription^17–21^. Additionally, MAPK pathways (i.e. ERK, JNK, p38) participate in the cellular response to microbial and inflammatory stimuli and contribute to *HBD2* expression^22,23^. TLRs signaling, crucial for recognizing microbial components, also activates downstream pathways influencing *HBD2* expression, like the PI3K/Akt pathway, known for its roles in immune responses^24^. Finally, the Jak/STAT pathway, involved in cytokine signaling, also contributes to the regulation of *HBD2*^25^.

Here, we brought out several host factors involved in signaling pathways, including receptors and proteins from signal transduction cascades (Fig. 3d, Supplementary Table S1, Supplementary Table S2). Among them, we found TLR5 and the downstream adaptor MYD88, whose engagement leads to NF-κB activation in response to a bacterial challenge^12,13^. We also identified LURAP1L and RBCK1, involved in the regulation of NF-κB signaling through ubiquitination of IKK subunits within the IKK complex ^26^. Regarding MAPK signaling, we found DOCK6 and MAPK14, exerting an antagonistic effect on *HBD2* expression. *DOCK6* encodes a member of the Dedicator of Cytokines (DOCK) family and is involved in ERK activation, whereas *MAPK14* encoding p38α contributes to MAPKAP protein activation through its phosphorylation^27,28^. Finally, we identified the NLRP6 and NLRC4 inflammasomes as belonging to the regulatory circuits controlling *HBD2* expression^29,30^. NLRP6, which acts as a cytosolic innate immune sensor that recognizes MAMPs, promotes *HBD2* expression, while NLRC4 would dampen it. Noteworthy, inflammatory bowel diseases in which *HBD2* expression is dysregulated may be associated with aberrant expression or mutations in these two genes^9,30,31^. Collectively, these intricate signaling networks would enable a finely tuned regulation of *HBD2*, allowing the immune system to mount effective responses against microbial threats.

At the transcriptional level, the involvement of key transcription factors such as NF-κB and AP-1 in *HBD2* expression emphasizes the adaptability of the immune response to diverse stimuli^32,33^. Complementing this, contributions of the CREB factors to the basal transcriptional activity of *HBD2* provide additional layers of complexity, suggesting a nuanced regulatory network that responds to both basal and induced immune states^34^. Besides response elements recognized by NF-κB, AP-1 and CREB, the fact that the *HBD2* promoter has a mosaic structure containing various transcriptional binding motifs suggests that several other transcription factors would participate in the regulation of this antimicrobial peptide gene, to fine-tuning its expression with regard to the immune context^15^.

In agreement with this genomic observation, our results revealed several new transcription factors involved in the control of *HBD2* expression (Supplementary Table S1, Supplementary Table S2). Among them, FIZ1, PITHD1, PURB, SCRT2, ZNF3, ZNF672 and ZNF821 promote *HBD2* transcription, whereas ATMIN, GATA6, HSFX1, MDM4, NCOA6, NFYC, NIBAN1, NPAS2, SFPQ, SRF, SUPT5H, ZC3H4, ZNF165 and ZNF558 inhibit it. These transcription factors may either belong to the primary transcriptional response, by binding to the *HBD2* promoter through a dedicated motif, or to the secondary response, by controlling the expression of another transcription factor which in turn, bind directly to the *HBD2* promoter. According to this scenario, targeted investigations on *ZNF3* and *GATA6*, by silencing their expression in human wild-type intestinal epithelial cells challenged with *E. coli*, confirmed their antagonistic role on *HBD2* expression (Fig. 5). Interestingly, our results are consistent with the increased *HBD2* mRNA expression and HBD2 peptide production measured in biopsies from ulcerative colitis patients, an IBD in which *GATA6* expression may be decreased^35,36^. Further studies using resolutive experimental approaches, including chromatin immuno-precipitation experiments, will allow to determine whether GATA6 orchestrates the modulation of *HBD2* expression directly, through its binding to the gene promoter region, or indirectly using a regulatory loop involving other factors.

Epigenetic modifications emerge as key regulatory mechanisms of *HBD2* expression. DNA methylation, a dynamic process in the promoter region, exerts control over *HBD2* accessibility and transcription^37,38^. This modification provides a long-lasting imprint on the *HBD2* gene, linking environmental cues to stable changes in its expression. Histone modifications, particularly acetylation, methylation and phosphorylation, act as dynamic regulators, influencing chromatin structure and, subsequently, *HBD2* expression^38–40^. These epigenetic marks contribute to the plasticity of *HBD2* regulation, allowing for rapid adaptation to varying immune challenges.

Here, we brought out several host factors involved in epigenetic regulations and associated molecular mechanisms. Among them, we identified BRD4 and JMJD6 as both inhibiting *HBD2* expression. *BRD4* encodes a bromodomain-containing protein and *JMJD6*, a jumonji-domain containing protein. Both are described to interact together and to co-bind enhancers to regulate promoters of transcription units^41^. Conversely, we identified *SUDS3* as a factor promoting *HBD2* transcription. Targeted validation by silencing *SUDS3* in wild-type intestinal epithelial cells confirmed much less transcription of *HBD2* upon a bacterial challenge (Fig. 5). Although the *SUDS3* gene encodes a protein which is mainly known to repress the gene transcription, deficiency of its expression may also lead in a loss of gene expression, as observed for the *NANOG* and *SOX2* genes both encoding transcription factors^42,43^. Deciphering mechanisms associated to these chromatin regulators would unravel the complexity of *HBD2* regulation, where a precise control is exerted to modulate its expression in response to varying immune requirements.

Beyond signaling pathways, transcription factors and epigenetic regulators, additional regulatory mechanisms further shape *HBD2* expression. MicroRNA-mediated post-transcriptional regulation adds yet another layer of sophistication to *HBD2* control. MIR146a, identified in the regulatory network, exemplifies how fine-tuning can occur at the post-transcriptional level, providing an additional level of control over *HBD2* expression in specific pathological context^44^.

Our work revealed a new microRNA, MIR9-1HG, potentially involved in the control of *HBD2*. Depletion of this host factor in intestinal epithelial cells increased *HBD2* expression (Supplementary Table S2). Further experiments, like the use of microRNA sponges binding MIR9-1HG, would be therefore required to confirm its impact on *HBD2* expression and abundance of this antimicrobial peptide. Validating involvement of this type of post-transcriptional control in the multi-leveled regulatory circuits of *HBD2* expression, would confirm the crucial role of microRNAs in the control of innate immune defense against microorganisms.

To conclude, given the extent, spread and impact of antimicrobial resistance in bacteria, new molecules and approaches are urgently needed in the anti-infective drug-discovery pipeline. This has stimulated interest in the use of antimicrobial peptides as therapeutic targets. The inducible nature of these peptides and the existence of regulatory circuits disconnecting their expression from proinflammatory mediators highlight the possibility of developing an innovative therapeutic strategy aiming at boosting their endogenous expression for the prevention or treatment of infections or diseases^39,45,46^. Despite false-positive or false-negative inherent to the genome-wide siRNA screening approach used, which were flagged by counter-screenings and targeted confirmations, this comprehensive study on the *HBD2* regulation provides valuable insights into the adaptability and precision of antimicrobial peptide expression. The dynamic interplay of signaling, transcriptional and epigenetic elements shows how *HBD2* can serve as a versatile component of the immune armamentarium, capable of fine-tuning its expression to different immune challenges. The knowledge gained from unraveling these regulatory networks in human intestinal epithelial cells not only enhances our understanding of mucosal innate immunity but also holds potential for the development of targeted therapeutic strategies in various clinical contexts. Continued research will likely unveil additional layers of complexity in *HBD2* regulation, but above all will pave the way for developing an innovative approach to modulate its expression for therapeutic purposes.

## Methods

### Cell culture

The human colonic epithelial cell line Caco-2, subclone TC7^47^, was cultured with DMEM (Thermo Fisher) supplemented with 10% (vol/vol) decomplemented FBS (Thermo Fisher), 1% nonessential amino acids (Thermo Fisher), 100 U/mL penicillin, and 100 μg/mL streptomycin (Thermo Fischer), at 37°C in 10% CO_2_. Cells were split two times per week using Versene solution (Thermo Fisher). When specified, 20 ng/mL gamma-interferon (R&D Systems), 20 ng/mL interleukin-17 (R&D Systems), 20 ng/mL interleukin-1-beta (R&D Systems), or 5 μg/mL flagellin (InvivoGen) were added to the complete media.

### Bacterial challenge

The *Escherichia coli* K12 (ATCC 12435) and *E. coli* LF82^48^ were isolated and grown in LB medium (Sigma) at 37°C. The *Streptococcus macedonicus*^49^ and *Streptococcus gallolyticus* (ATCC 9808) were isolated and grown in BHI medium (Sigma) at 37°C. For bacterial challenge, cells were grown at confluence in 6-well plates (1.5 × 10^6^ cells per well) for 48 h at 37°C and 10% CO_2_. Challenges of cells were performed using complete DMEM without antibiotics, using overnight bacterial cultures, at a multiplicity of infection (MOI) ranging from 1 to 100 bacteria per cell, from 1 to 6 h. When required, bacterial challenges were stopped with 100 U/mL penicillin and 100 μg/mL streptomycin (Thermo Fischer).

### RNA extraction and qRT-PCR experiment

RNA was isolated using the RNeasy Mini kit and the RNase free DNase kit (Qiagen). RT-PCR reactions were performed overnight using the SuperScript II reverse transcriptase (Thermo Fisher) and the oligo(dT)18primers (Thermo Fisher), as recommended by the supplier. Gene-specific primers were designed and purchased from Sigma (*HBD2*, GCCATGAGGGTCTTGTATCTC/TTAAGGCAGGTAACAGGATCG; e*GFP*, AGCCACAACGTCTATATCATGG/GGTGTTCTGCTGGTAGTGGTC). The qRT-PCR reactions were carried out in a 20 μL final volume containing 8 μL of cDNA (diluted at 1/100), 2 μL of primers (0.2 μM each), and 10 μL of Power SYBR Green PCR Master Mix (Thermo Fisher). Reactions were run on a QuantStudio 7 PCR system (Thermo Fisher) with the recommended universal thermal cycling parameters. Each sample reaction was run in duplicate on the same plate. Relative gene-expression quantification was performed using the comparative cycle threshold (Ct) method. Data were normalized using the beta-2-microglobulin housekeeping gene (*B2M*, ATTGCTATGTGTCTGGGTTTCA/AAGACAAGTCTGAATGCTCCAC).

### ELISA assay

We used the ELISA kit for HBD2 (900-K172, PeproTech), as recommended by the supplier. Absorbance was measured on a M200PRO fluorimeter (Tecan).

### Stable *HBD2* reporter cell line engineering

A stable TC7 cell line expressing the eGFP reporter gene under the control of the *HBD2* gene promoter was constructed using the ViraPower Lentiviral Gateway Expression kit, as recommended by the supplier (Thermo Fisher). Briefly, the *HBD2* promoter (3.5 kb upstream ATG of the *HBD2* gene, also known *DEFB4A*, ID1673) was amplified from TC7 cell genomic DNA using the PHUSION High-Fidelity DNA Polymerase (Thermo Fisher) with primers from Sigma matching the DNA region (CACTGTACCACCAGTAGCAATAACCG/CTGATGGCTGGGAGCTTCACCAGGAG), and cloned into the pENTR5’-TOPO vector (Thermo Fisher). The eGFP reporter gene (enhanced Green Fluorescent Protein) was amplified from the pEGFP-LC3 plasmid (Addgene) using the TaKaRa Taq DNA Polymerase (Takara) with primers from Sigma matching the eGFP gene (CACCATGGTGAGCAAGGGCGAGGAG/TTAAGATACATTGATGAGTTTGGAC), and cloned into the pENTR vector (Thermo Fisher). Both vectors were fused to the pLenti6/R4R2/V5-DEST vector to get the pLenti6/R4R2/V5-DEST *HBD2*-eGFP expression construct. Expression plasmid and optimized ViraPower packaging mix were used to co-transfect the ViraPower 293FT producer cell line, using Lipofectamine 2000 reagent and Opti-MEM I reduced serum medium (Thermo Fisher). Cell supernatant containing lentivirus was harvested, titrated using the crystal violet method, and used with polybrene (Sigma) to transduce wild-type TC7 cells. Several clones were selected on blasticidin, isolated using limit dilutions, individually grown, and assayed for inducible expression of the eGFP upon stimulation with cytokines or inducers of *HBD2*, like gamma-interferon (R&D Systems), interleukin-17 (R&D Systems), interleukin-1-beta (R&D Systems) or flagellin (InvivoGen). Further characterizations were carried out on clones to select the most representative one, including cell morphology analysis and integration site determination. The clone used in this work (i.e. clone 6.1) is available at the Collection Nationale de Cultures de Microorganismes (CNCM) from Institut Pasteur (Paris, France), under the reference number CNCM I-4536.

### Epifluorescence GFP signal detection

To assess the functionality of transcriptional fusions between *HBD2* promoter and eGFP, detection of the GFP signal in stable TC7 reporter clones was assayed as follows. Cell monolayers of clones were grown at confluence on coverslips in 12-well plates (0.75 × 10^6^ cells per well) for 48 h, and stimulated or not with the flagellin *HBD2* inducer for 24 h, using 5 μg/mL final concentration. Cells were washed with PBS and fixed with Carnoy reagent (60% ethanol, 30% acetic acid, 10% chloroform), at room temperature. After a washing step, coverslips were recovered and mounted using 1% DABCO mounting medium (Sigma-Aldrich), sealed and dried overnight at room temperature before examination. GFP signal was detected by epifluorescence microscopy using 40 × magnification (Zeiss).

### Human genome siRNA library

The Human ON-Target plus SMARTpool siRNA library targeting 18,236 unique human genes was purchased from Dharmacon (Thermo Fischer) at the 384-well plate format dispatched in 58 plates, at 1 μM final concentration. The collection was delivered in three parts: core druggable subsets (9 plates), drug targets (15 plates), remaining genome collection (34 plates).

### Whole human genome siRNA screening

The human pan-genomic siRNA screening was performed in 384-well plates from Greiner (Sigma-Aldrich). Clone 6.1 was transfected either with scrambled or GFP control siRNA, or the human genome siRNA library. Briefly, cells were dissociated with Versene solution (Thermo Fischer) one day prior to transfection, diluted in fresh DMEM medium (Thermo Fischer) supplemented with 10% FBS (Thermo Fischer), 1% nonessential amino acids (Thermo Fisher) without antibiotics, and transferred into 384-well plates. A total of 8000 cells were seeded per well and cultured at 37°C for 16 h, under 10% CO_2_. Transient transfection of siRNAs was performed using DharmaFECT 4 transfection reagent (Thermo Fischer). For each well, 4.95 μL of serum-free DMEM and 0.05 μL of DharmaFECT 4 were preincubated for 5 min at room temperature. At the same time, 2.5 μL of serum-free DMEM was mixed with 2.5 μL of each siRNA (1 μM) and incubated for 5 min at room temperature. The two mixtures were combined and incubated for 20 min at room temperature, to allow complex formation. After addition of 40 μL of complete DMEM medium without antibiotics to the mixture, the entire solution was added in each well onto the cells, resulting in a final concentration of 50 nM for each siRNA. After the transfection step, cells were incubated for 48 h to allow gene silencing. Cells were then challenged with *E. coli* K12 at a MOI of 10 bacteria per cell (50 μL/well) for 3 h, and washed out to remove bacteria. Cells were subsequently incubated in fresh complete DMEM with 100 U/mL penicillin and 100 μg/mL streptomycin (Thermo Fischer), for additional 24 h. Cells were finally fixed in 4% paraformaldehyde (Sigma-Aldrich) at room temperature and stained with 2.5 μM DRAQ5 (BioStatus), before imaging. The whole human genome siRNA screening was performed in duplicate, in two independent experiments.

### siRNA specificity confirmation

To exclude possible off-target effects due to pooled siRNA duplexes in the library, a validating counter-screening using single siRNA duplexes from Dharmacon (Thermo Fischer) was performed on 57 genes identified in the primary screening. For these experiments, all procedures were performed in exactly the same way than described for the whole human genome siRNA screening, except that 4 single siRNA duplexes were individually tested to silence each gene. The counter-screening was performed in four independent experiments.

### Image acquisition and analysis

The siRNA library screening images were acquired on an automated confocal microscope OPERA QEHS (PerkinElmer) with a 10 × objective in the following sequence: GFP signal (ex/em 484/510), nucleus detection with DRAQ5 (ex/em 640/690). A field of 10 adjacent images was acquired per well, covering ≈50% of each measured 384-well plate. Images were analyzed using the Acapella software (PerkinElmer). Cell scoring parameters were determined to evaluate the proportion of GFP expressing cells, which was calculated according to the fluorescence intensity and nuclei numbers. The results were normalized to the total amount of cells in the image and converted into the percentage of GFP expressing cells.

### Statistics

GraphPad Prism 10 (GraphPad software) was used to determine statistical significance for comparison of two groups by one-way analysis of variance (ANOVA) and the Bonferroni method. A *P* value < 0.05 was considered statistically significant.

### Supplementary Information

Supplementary Information.

## Data Availability

Data that support the finding of this study are available from the corresponding author upon request.
