# Supplementary material for: Identification of human host factors required for beta-defensin-2 expression in intestinal epithelial cells upon a bacterial challenge
Source: Sci Rep. 2024 Jul 4;14:15442. doi: 10.1038/s41598-024-66568-y (PMC11224401; doi:10.1038/s41598-024-66568-y)
Supplement: Supplementary file 1 — Supplementary Information. [file 41598_2024_66568_MOESM1_ESM.pdf]

Identification of human host factors required for beta-defensin-2 expression in intestinal epithelial cells upon a bacterial challenge

Weronika Wozniak[1], Emmanuel Sechet[2], Yong-Jun Kwon[3], Nathalie Aulner[2], Lionel Navarro[1] & Brice Sperandio[1]

[1]Institut de Biologie de l'École Normale Supérieure (IBENS), Université PSL, Centre National de la Recherche Scientifique (CNRS) UMR8197, Institut National de la Santé et de la Recherche Médicale (INSERM) U1024, Paris, France.
[2]Institut Pasteur, Université Paris Cité, Paris, France.
[3]Institut Pasteur Korea, Seoul, South Korea. Present address: Luxembourg Institute of Health, Dudelange, Luxembourg.

Supplementary Table S1. List of the human genes promoting expression of the beta-defensin *HBD2* gene.

Gene*	Gene name*	GeneID*	Subcellular location#	Molecular function#	Biological process#
**AGAP1**	ArfGAP with GTPase domain, ankyrin repeat and PH domain 1	116987	Cytoplasm	GTPase activation	Protein transport
**ANO10**	Anoctamin 10	55129	Cell membrane	Ion channel activity	Ion transmembrane transport
**B3GAT2**	Beta-1,3-glucuronyltransferase 2	135152	Unknown	Oxydoreductase	Glycolysis
**BRI3BP**	BRI3 binding protein	140707	Mitochondrion	Protein stabilizing	Tumorigenesis
**BRINP2**	BMP/retinoic acid inducible neural specific 2	57795	Secreted	Unknown	Cell cycle
**C1orf105**	Chromosome 1 open reading frame 105	92346	Unknown	Unknown	Unknown
**C2CD3**	C2 domain containing 3 centriole elongation regulator	26005	Centriole, cytoplasms	Positive regulator	Centriole elongation
**C2orf42**	Chromosome 2 open reading frame 42	54980	Nucleus	Unknown	Unknown
**CALU**	Calumenin	813	Endoplasmic reticulum, Golgi apparatus	Calcium binding	Carboxylation
**CCDC42**	Coiled-coil domain containing 42	146849	Unknown	Unknown	Spermatid development
**CD37**	CD37 molecule	951	Membrane	Protein binding	Signal transduction
**CDH10**	Cadherin 10	1008	Cell membrane	Metal binding	Cell adhesion
**CLEC4D**	C-type lectin domain family 4 member D	338339	Cell membrane	Metal binding	Immunity
**CPNE2**	Copine 2	221184	Cytoplams, nucleus	Calcium binding	Calcium response
**CPSF6**	Cleavage and polyadenylation specific factor 6	11052	Nucleus, cytoplasm	RNA binding	mRNA processing
**CRYGS**	Crystallin gamma S	1427	Unknown	Eye lens protein	Epithelium morphogenesis
**CTAG1B**	Cancer/testis antigen 1B	1485	Cytoplasm	Protein binding	tRNA metabolic process
**DENND1B**	DENN domain containing 1B	54530	Cytoplasm	Guanine-nucleotide releasing factor	Protein transport
**DNAI2**	Dynein axonemal intermediate chain 2	64446	Cytoplasm, cytoskeleton	Motor protein	Cilium biogenesis
**DOK6**	Docking protein 6	220164	Cytoplasm	Adaptor	Positive regulation of MAPK
**EBLN2**	Endogenous Bornavirus like nucleoprotein 2	55096	Unknown	RNA binding	Unknown
**ECHDC2**	Enoyl-CoA hydratase domain containing 2	55268	Mitochondrion	Lyase	Lipid metabolism
**EDC3**	Enhancer of mRNA decapping 3	80153	Cytoplasm	RNA binding	mRNA degradation
**EEF1AKMT1**	EEF1A lysine methyltransferase 1	221143	Cytoplasms	Methyltransferase	Methylation
**FBLN2**	Fibulin 2	2199	Secreted	Fibronectin binding	Extracellular matrix organization
**FCN3**	Ficolin 3	8547	Secreted	Lectin binding	Innate immunity
**FIZ1**	FLT3 interacting zinc finger 1	84922	Cytoplasm, nucleus	Repressor	Transcription regulation
**FJX1**	Four-jointed box kinase 1	24147	Secreted	Dendrite extension inhibitor	Cell-cell signaling
**FTMT**	Ferritin mitochondrial	94033	Mitochondrion	Oxidoreductase	Iron storage
**IFT88**	Intraflagellar transport 88	8100	Cytoplasm	Unknown	Cilium biogenesis
**IGLL3P**	Immunoglobulin lambda like polypeptide 3	91353	Unknown	Pseudogene	Unknown
**IQCE**	IQ motif containing E	23288	Cell projection	Positive regulation of Hedgehog signaling	Limb morphogenesis

**ISCA2**	Iron-sulfur cluster assembly 2	122961	Mitochondrion	Iron binding	Protein maturation
**KLHDC7A**	Kelch domain containing 7A	127707	Membrane	Unknown	Unknown
**KRI1**	KRI1 homolog	65095	Nucleolus	RNA binding	Endonucleolytic cleavage
**LINC02904**	Long intergenic non-protein coding RNA 2904	286122	Unknown	Noncoding RNA	Unknown
**LURAP1L**	Leucine rich adaptor protein 1 like	286343	Unknown	Positive regulation of I-kappaB kinase	NF-kappaB signaling
**LYRM1**	LYR motif containing 1	57149	Nucleus	Unknown	Cell proliferation
**MAB21L2**	Mab-21 like 2	10586	Nucleus, cytoplasm	Developmental protein	Cell proliferation
**MPRIP**	Myosin phosphatase Rho interacting protein	23164	Cytoskeleton	Actin binding	Actin organization
**MYD88**	MYD88 innate immune signal transduction adaptor	4615	Cytoplams, nucleus	Receptor binding	Innate immunity
**NHLRC3**	NHL repeat containing 3	387921	Secreted	Ubiquitin protein ligase activity	Protein catabolic process
**NIT2**	Nitrilase family member 2	56954	Cytoplasm	Hydrolase	Metabolic process
**NLRP6**	NLR family pyrin domain containing 6	171389	Inflammasome, cytoplasm	Binding	Innate immunity
**ORAI2**	ORAI calcium release-activated calcium modulator 2	80228	Membrane	Calcium channel activity	Calcium entry
**PCP4**	Purkinje cell protein 4	5121	Cytoplams, nucleus	Calmodulin binding	Calmodulin signalling pathway
**PDZK7**	PDZ domain containing 7	79955	Nucleus, cell projection	Protein binding	Protein localization
**PITHD1**	PITH domain containing 1	57095	Cytoplasm	Activator	Transcription regulation
**PLEKHA8**	Pleckstrin homology domain containing A8	84725	Golgi apparatus	Lipid binding	Lipid transport
**POU2AF2**	POU class 2 homeobox associating factor 2	341032	Unknown	Unknown	Unknown
**PPP1R35**	Protein phosphatase 1 regulatory subunit 35	221908	Cytoplasm	Protein phosphatase inhibitor	Negative regulation of phosphatase activity
**PPP1R36**	Protein phosphatase 1 regulatory subunit 36	145376	Unknown	Protein phosphatase inhibitor	Negative regulation of phosphatase activity
**PTGFRN**	Prostaglandin F2 receptor inhibitor	5738	Endoplasmic reticulum, Golgi apparatus	Inhibitor of prostaglandin F2-alpha	Lipid droplet organization
**PUM3**	Pumilio RNA binding family member 3	9933	Nucleus	DNA/RNA binding	Translation regulation
**PURB**	Purine rich element binding protein B	5814	Nucleus	Repressor	Transcription regulation
**RBCK1**	RANBP2-type and C3HC4-type zinc finger containing 1	10616	Cytosol	Transferase	NF-kappaB signaling
**RBM42**	RNA binding motif protein 42	79171	Cytoplams, nucleus	RNA binding	mRNA splicing regulation
**SCRT2**	Scratch family transcriptional repressor 2	85508	Nucleus	DNA binding	Transcription regulation
**SECTM1**	Secreted and transmembrane 1	6398	Cell membrane, secreted	Cytokine activity	Immune response
**SERPINH1**	Serpin family H member 1	871	Endoplasmic reticulum	Chaperone	Stress response
**SH3TC1**	SH3 domain and tetratricopeptide repeats 1	54436	Plasma membrane, recycling endosome	Unknown	Regulation of ERBB signaling pathway
**SMC5**	Structural maintenance of chromosomes 5	23137	Nucleus	Nuclotide binding	DNA damage
**SMG9**	SMG9 nonsense mediated mRNA decay factor	56006	Cytosol	Protein binding	Nonsense-mediated mRNA decay
**SRGN**	Serglycin	5552	Cytoplasmic granule, Golgi apparatus	Unknown	Secretory granule

**SST**	Somatostatin	6750	Secreted	Hormone	Signaling
**SUDS3**	SDS3 homolog, SIN3A corepressor complex component	64426	Nucleus	Chromatin regulator	Transcription regulation
**TAS2R39**	Taste 2 receptor member 39	259285	Membrane	Receptor	Sensory transduction
**TENT5B**	Terminal nucleotidyltransferase 5B	115572	Cytoplams, nucleus	Nucleotidyltransferase	mRNA stabilization
**THADA**	THADA armadillo repeat containing	63892	Endoplasmic reticulum	Unknown	Apoptosis
**TIMM23**	Translocase of inner mitochondrial membrane 23	10431	Mitochondrion	Protein transporter	Protein transport
**TLR5**	Toll like receptor 5	7100	Cell membrane	Receptor	Innate immunity
**TMUB1**	Transmembrane and ubiquitin like domain containing 1	83590	Membrane, cytoplasm, nucleus	Transmembrane protein	Ubiquitin-dependent ERAD pathway
**UBALD2**	UBA like domain containing 2	283991	Unknown	Unknown	Unknown
**UBXN11**	UBX domain protein 11	91544	Cytoplasm, cytoskeleton	Ubiquitin binding	Protein catabolic process
**UTRN**	Utrophin	7402	Cytoplasm, cytoskeleton	Actin binding	Cytoskeleton anchoring
**ZC3H18**	Zinc finger CCCH-type containing 18	124245	Nucleus	RNA binding	RNA destabilization
**ZNF3**	Zinc finger protein 3	7551	Nucleus	DNA binding	Transcription regulation
**ZNF672**	Zinc finger protein 672	79894	Nucleus	DNA binding	Transcription regulation
**ZNF821**	Zinc finger protein 821	55565	Nucleus	DNA binding	Transcription regulation

*Data from Gene (https://www.ncbi.nlm.nih.gov/gene/).

#Data from UniProtKB (https://www.uniprot.org/uniprotkb).

Supplementary Table S2. List of the human genes inhibiting expression of the beta-defensin *HBD2* gene.

Gene*	Gene name*	GeneID*	Subcellular location[#]	Molecular function[#]	Biological process[#]
**ADPRM**	ADP-ribose/CDP-alcohol diphosphatase, manganese dependent	56985	Cytosol	Hydrolase	Immune cell signaling
**APOD**	Apolipoprotein D	347	Secreted	Lipid binding	Lipid transport
**AQP5**	Aquaporin 5	362	Cell membrane	Water channel activity	Transport
**AREG**	Amphiregulin	374	Membrane	Growth factor	EGFR ligand
**ARL4D**	ADP ribosylation factor like GTPase 4D	379	Nucleus, cytoplasm	Nucleotide binding	Protein transport
**ATMIN**	ATM interactor	23300	Nucleus	Activator	Transcription regulation
**AUNIP**	Aurora kinase A and ninein interacting protein	79000	Nucleus, chromosome	DNA binding	DNA repair
**AWAT2**	Acyl-CoA wax alcohol acyltransferase 2	158835	Endoplasmic reticulum	Acyltransferase	Lipid metabolism
**BRD4**	Bromodomain containing 4	23476	Nucleus, chromosome	Chromatine regulator	Transcription regulation
**C17orf78**	Chromosome 17 open reading frame 78	284099	Membrane	Unknown	Unknown
**C7orf50**	Chromosome 7 open reading frame 50	84310	Unknown	RNA binding	Unknown
**CAB39**	Calcium binding protein 39	51719	Cytoplasm	Kinase binding	Activation of protein kinase activity
**CAPN6**	Calpain 6	827	Cytoplasm	Microtubule binding	Cytoskeleton organization
**CD79A**	CD79a molecule	973	Cell membrane	Protein binding	Adaptive immunity
**CD99L2**	CD99 molecule like 2	83692	Cell membrane	Adhesion protein	Cell adhesion
**CEACAM7**	CEA cell adhesion molecule 7	1087	Cell membrane	Glycoprotein	Unknown
**COX6C**	Cytochrome c oxidase subunit 6C	1345	Mitochondrion	Component of the cytochrome c oxydase	Cellular respiration
**CPSF1**	Cleavage and polyadenylation specific factor 1	29894	Nucleus	RNA binding	mRNA processing
**CRACR2B**	Calcium release activated channel regulator 2B	283229	Cytoplasm	Ion binding	Store-operated calcium entry
**CRIPAK**	Cysteine rich PAK1 inhibitor	285464	Nucleus, cytoplasm	Repressor	PAK1 regulation
**CSK**	C-terminal Src kinase	1445	Cytoplasm, membrane	Kinase	Adaptive immunity
**CYB5R1**	Cytochrome b5 reductase 1	51706	Membrane	Oxydoreductase	Lipid metabolism
**DDI1**	DNA damage inducible 1 homolog 1	414301	Unknown	Aspartyl protease	Regulation of protein stability
**DSCR10**	Down syndrome critical region 10	259234	Unknown	ncRNA	Unknown
**EEF1B2**	Eukaryotic translation elongation factor 1 beta 2	1933	Cytoplasm	Elongation factor	Protein biosynthesis
**EIF4A3**	Eukaryotic translation initiation factor 4A3	9775	Nucleus, cytoplasm	RNA binding	mRNA processing
**ELSPBP1**	Epididymal sperm binding protein 1	64100	Secreted	Heparin binding	Fertilization
**ENHO**	Energy homeostasis associated	375704	Secreted	Hormone activity	Positive regulation of Notch signaling pathway
**ERCC8**	ERCC excision repair 8, CSA ubiquitin ligase complex subunit	1161	Nucleus	Protein-containing complex binding	DNA repair
**FBLN5**	Fibulin 5	10516	Secreted	Protein binding	Cell adhesion
**FCGR2B**	Fc gamma receptor IIb	2213	Cell membrane	IgG binding	Immune response
**GATA6**	GATA binding protein 6	2627	Nucleus	Activator	Transcription regulation

**HSFX1**	Heat shock transcription factor family, X linked 1	51402	Nucleus, cytoplasm	DNA binding	Transcription regulation
**IGSF11**	Immunoglobulin superfamily member 11	152404	Cell membrane	Receptor	Cell adhesion
**IMPG1**	Interphotoreceptor matrix proteoglycan 1	3617	Secreted	Receptor	Extracellular matrix organization
**INTS10**	Integrator complex subunit 10	55174	Nucleus	Component of the Integrator complex	snRNA processing
**INTS13**	Integrator complex subunit 13	55726	Nucleus, cytoplasm	Component of the Integrator complex	Cell cycle
**INTS5**	Integrator complex subunit 5	80789	Nucleus, cytoplasm	Component of the Integrator complex	snRNA processing
**INTS6**	Integrator complex subunit 6	26512	Nucleus	Component of the Integrator complex	snRNA processing
**INTS7**	Integrator complex subunit 7	25896	Nucleus, cytoplasm	Integrator complex protein	DNA damage
**JMJD6**	Jumonji domain containing 6, arginine demethylase and lysine hydroxylase	23210	Nucleus, cytoplasm	Chromatine regulator	Transcription regulation
**JUP**	Junction plakoglobin	3728	Membrane	Protein binding	Cell adhesion
**KIF11**	Kinesin family member 11	3832	Cytoplasm	Motor protein	Cell cycle
**KRT33B**	Keratin 33B	3884	Cytosol, cytoskeleton	Structural molecule activity	Epithelial cell differentiation
**LIN7B**	Lin-7 homolog B, crumbs cell polarity complex component	64130	Cell membrane	Protein domain specific binding	Protein transport
**LYSMD3**	LysM domain containing 3	116068	Membrane, Golgi apparatus	Membrane protein	Golgi organization
**MAPK14**	Mitogen-activated protein kinase 14	1432	Nucleus, cytoplasm	Kinase	Transcription regulation
**MARCHF4**	Membrane associated ring-CH-type finger 4	57574	Golgi apparatus	Transferase	Ubl conjugation pathway
**MDC1**	Mediator of DNA damage checkpoint 1	9656	Nucleus, chromosome	Protein binding	DNA repair
**MDM4**	MDM4 regulator of p53	4194	Nucleus	p53 binding	p53 regulation
**METTL15**	Methyltransferase like 15	196074	Mitochondrion	Methyltransferase	rRNA base methylation
**METTL5**	Methyltransferase 5, N6-adenosine	29081	Nucleus	Methyltransferase	rRNA methylation
**MFSD3**	Major facilitator superfamily domain containing 3	113655	Membrane	Proton symporter activity	Transport
**MIR9-1HG**	MIR9-1 host gene	10485	Nucleus	ncRNA	Transcription regulation
**MTMR3**	Myotubularin related protein 3	8897	Cytoplasm	Protein phosphatase	Lipid metabolism
**NAA11**	N-alpha-acetyltransferase 11, NatA catalytic subunit	84779	Nucleus, cytoplasm	Acyltransferase	Protein amino acid acetylation
**NAPB**	NSF attachment protein beta	63908	Membrane	Protein binding	Protein transport
**NCOA6**	Nuclear receptor coactivator 6	23054	Nucleus	Activator	Transcription regulation
**NFYC**	Nuclear transcription factor Y subunit gamma	4802	Nucleus	DNA binding	Transcription regulation
**NIBAN1**	Niban apoptosis regulator 1	116496	Cytoplasm, membrane	Unknown	Translational regulation
**NLRC4**	NLR family CARD domain containing 4	58484	Cytoplasm, inflammasome	Protein binding	Innate immunity
**NPAS2**	Neuronal PAS domain protein 2	4862	Nucleus	Activator	Transcription regulation
**NSUN7**	NOP2/Sun RNA methyltransferase family member 7	79730	Unknown	Methyltransferase activity	Methylation
**OR2F1**	Olfactory receptor family 2 subfamily F member 1	26211	Cell membrane	G-protein coupled receptor	Sensory transduction
**OR5P2**	Olfactory receptor family 5 subfamily P member 2	120065	Cell membrane	G-protein coupled receptor	Sensory transduction
**PDHA2**	Pyruvate dehydrogenase E1 subunit alpha 2	5161	Mitochondrion	Oxydoreductase	Carbohydrate metabolism

**PGAM5**	PGAM family member 5, mitochondrial serine/threonine protein phosphatase	192111	Mitochondrion	Phosphatase activity	Necrosis
**PHF23**	PHD finger protein 23	79142	Nucleus, cytoplasm	Ion binding	Autophagy regulation
**PNPLA3**	Patatin like phospholipase domain containing 3	80339	Membrane, lipid droplet	Acyltransferase	Lipid metabolism
**POLR2B**	RNA polymerase II subunit B	5431	Nucleus	Nucleotidyltransferase	Transcription
**POLR2E**	RNA polymerase II, I and III subunit E	5434	Nucleus	DNA binding	Transcription
**POLR2F**	RNA polymerase II, I and III subunit F	5435	Nucleus	DNA binding	Transcription
**POLR2G**	RNA polymerase II subunit G	5436	Nucleus	RNA binding	Transcription
**POLR2H**	RNA polymerase II, I and III subunit H	5437	Nucleus	DNA binding	Transcription
**POLR2I**	RNA polymerase II subunit I	5438	Nucleus	Nucleic acid binding	Transcription
**POLR2L**	RNA polymerase II, I and III subunit L	5441	Nucleus	DNA binding	Transcription
**POMP**	Proteasome maturation protein	51371	Nucleus, cytoplasm	Chaperone	Proteasome assembly
**PRORSD1P**	Prolyl-tRNA synthetase associated domain containing 1, pseudogene	344405	Unknown	Aminoacyl-tRNA editing activity	Unknown
**PRXL2A**	Peroxiredoxin like 2A	84293	Cytoplasm, secreted	Antioxydant	Regulation of differentiation
**RAN**	RAN, member RAS oncogene family	5901	Nucleus, cytoplasm	Hydrolase	Protein transport
**RBM22**	RNA binding motif protein 22	55696	Nucleus, cytoplasm	RNA binding	mRNA splicing
**RNF115**	Ring finger protein 115	27246	Cytoplasm	Transferase	Ubl conjugation pathway
**SCD**	Stearoyl-CoA desaturase	6319	Endoplasmic reticulum	Oxydoreductase	Lipid metabolism
**SENP2**	SUMO specific peptidase 2	59343	Nucleus, cytoplasm	Protease	Transport
**SFPQ**	Splicing factor proline and glutamine rich	6421	Nucleus, cytoplasm	DNA binding	Transcription regulation
**SGTA**	Small glutamine rich tetratricopeptide repeat co-chaperone alpha	6449	Nucleus, cytoplasm	Chaperone	Endoplasmic reticulum targeting
**SIGLEC12**	Sialic acid binding Ig like lectin 12	89858	Membrane	Sialic acid binding	Cell adhesion
**SLBP**	Stem-loop binding protein	7884	Nucleus, cytoplasm	RNA binding	mRNA processing
**SMIM14**	Small integral membrane protein 14	201895	Endoplasmic reticulum	Membrane protein	Unknown
**SOS1**	SOS Ras/Rac guanine nucleotide exchange factor 1	6654	Cytoplasm	Guanine-nucleotide releasing factor	MAPK regulation
**SPIRE2**	Spire type actin nucleation factor 2	84501	Cytosol, cytoskeleton	Actin binding	Protein transport
**SPRR2E**	Small proline rich protein 2E	6704	Cytoplasm	Structural molecule activity	Keratinization
**SRF**	Serum response factor	6722	Nucleus	Activator	Transcription regulation
**STAR**	Steroidogenic acute regulatory protein	6770	Mitochondrion	Lipid binding	Lipid transport
**STARD3**	StAR related lipid transfer domain containing 3	10948	Late endosome membrane	Lipid binding	Lipid transport
**STIM1**	Stromal interaction molecule 1	6786	Cell membrane, endoplasmic reticulum	Ion binding	Ion transport
**SULT1C2**	Sulfotransferase family 1C member 2	6819	Cytoplasm, lysosome	Sulfotransferase	Sulfation
**SUPT5H**	SPT5 homolog, DSIF elongation factor subunit	6829	Nucleus	Regulator	Transcription regulation
**SURF2**	Surfeit 2	6835	Nucleoplasm, plasma membrane	Unknown	Unknown
**TAF2**	TATA-box binding protein associated factor 2	6873	Nucleus	Chromatine binding	Transcription regulation
**TMEM39A**	Transmembrane protein 39A	55254	Endoplasmic reticulum	Integral component of membrane	Autophagy regulation

**TRIM75**	Tripartite motif containing 75	391714	Cytoplasm	Zinc binding	Regulation of gene expression
**UQCRQ**	Ubiquinol-cytochrome c reductase complex III subunit VII	27089	Mitochondrion	Oxydoreductase	Electron transport
**USB1**	U6 snRNA biogenesis phosphodiesterase 1	79650	Nucleus	Nuclease	snRNA processing
**VIM**	Vimentin	7431	Nucleus, cytoplasm	Protein binding	Filament organization
**VIPR1**	Vasoactive intestinal peptide receptor 1	7433	Cell membrane	G-protein coupled receptor	VIP receptor
**WDR83OS**	WD repeat domain 83 opposite strand	51398	Endoplasmic reticulum	Chaperone	Protein insertion into ER membrane
**ZC3H4**	Zinc finger CCCH-type containing 4	23211	Chromosome	RNA binding	Transcription regulation
**ZNF165**	Zinc finger protein 165	7718	Nucleus	DNA binding	Transcription regulation
**ZNF558**	Zinc finger protein 558	148156	Nucleus	DNA binding	Transcription regulation

*Data from Gene (https://www.ncbi.nlm.nih.gov/gene/).
[#]Data from UniProtKB (https://www.uniprot.org/uniprotkb).

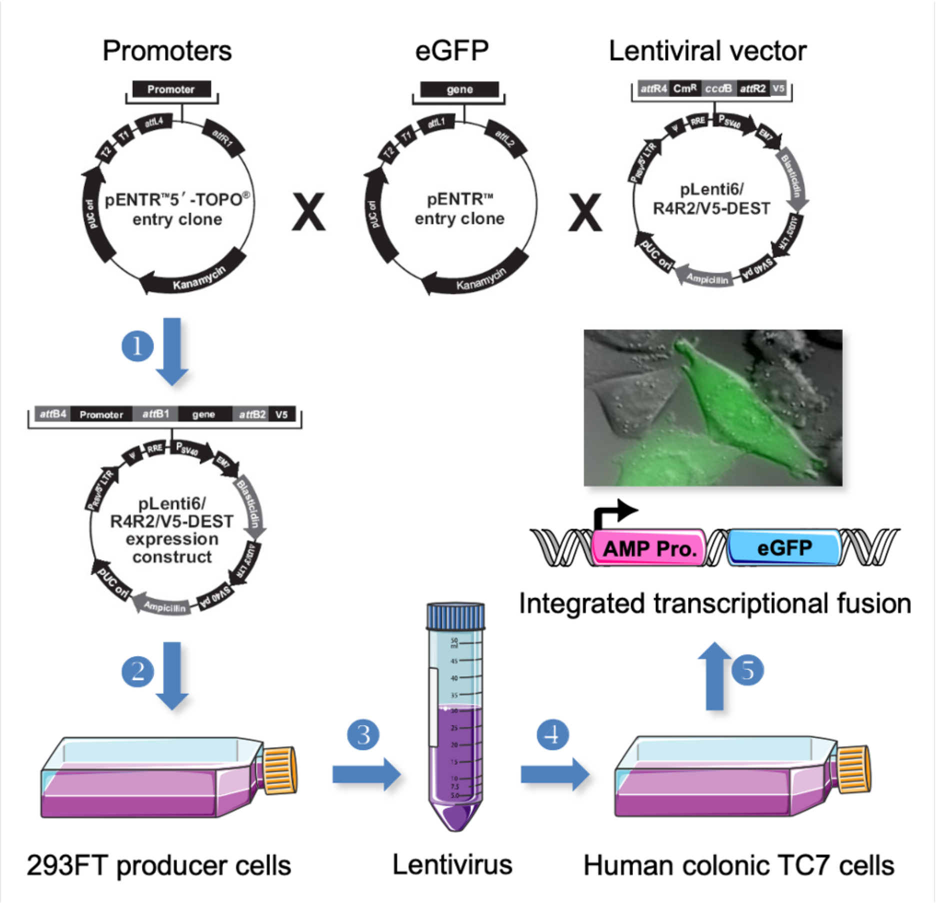

Supplementary figure S1: Construction of stable human intestinal epithelial TC7 cells reporting *HBD2* expression. The *HBD2* promoter (3.5 kb) was cloned into the pENTR5'-TOPO entry vector. The eGFP reporter gene was cloned into the pENTR entry vector. (1) The two plasmids were combined with the pLenti6/R4R2/V5-DEST lentiviral vector to get the pLenti6/R4R2/V5-DEST expression construct harboring the *HBD2* promoter-eGFP transcriptional fusion. (2) The mega-plasmid was transfected in 293FT producer cells allowing packaging of the transcriptional fusion into lentiviral capsides. (3) Cell supernatant containing lentivirus harboring the reporter construction was collected and the viral titer was determined. (4) Lentivirus were transduced in wild-type human intestinal epithelial TC7 cells at a multiplicity of infection of 1 to allow for a single lentiviral integration event per genome. (5) Blasticidin-resistant cells were isolated and cloned before to be characterized, as reported in Fig. 2.

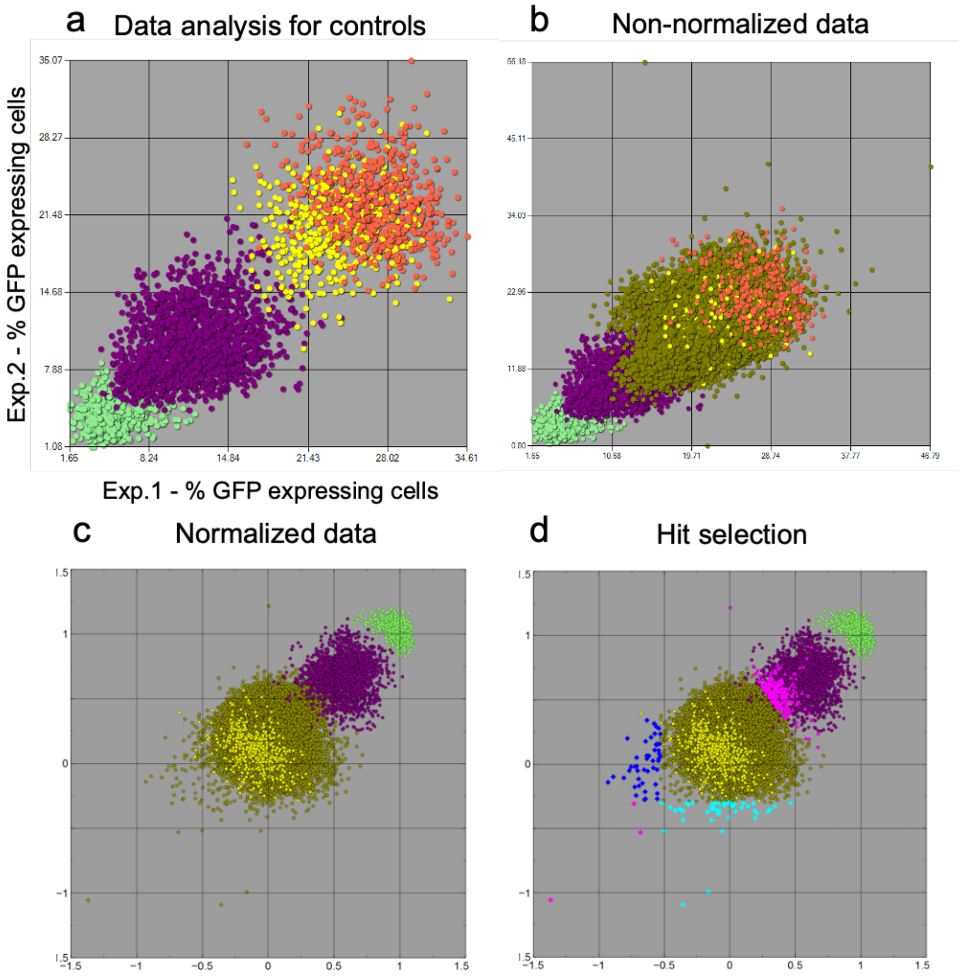

Supplementary figure S2: Dot-plot analysis of genome wide screening data coming from two independent experiments. Percentage of GFP expressing cells from experiments 1 (Exp. 1) and 2 (Exp.2) is reported on X and Y axis. (a) Analysis of data from siRNA controls. (b) Analysis of data from siRNA controls and siRNA library (non-normalized). (c) Analysis of data from siRNA controls and siRNA library (normalized). (d) Selection of 79 hits promoting *HBD2* expression (inhibiting GFP signal, pink triangles, cut-off $P > 0.25$), and 110 hits inhibiting *HBD2* expression (promoting GFP signal, pink/light blue/dark blue squares, cut-off $P < 0.005$). Robustness analysis Z' Factor = 0.5. Correlation coefficient > 0.8. Purple dots: non-transfected and non-challenged cells; yellow dots: non-transfected cells challenged with *E. coli*; orange dots: cells transfected with scramble siRNA and challenged with *E. coli*; light green dots: cells transfected with GFP siRNA and challenged with *E. coli*; dark green dots: cells transfected with the siRNA library and challenged with *E. coli*. Hit genes promoting *HBD2* expression are presented in Supplementary Table S1. Hit genes inhibiting *HBD2* expression are presented in Supplementary Table S2.

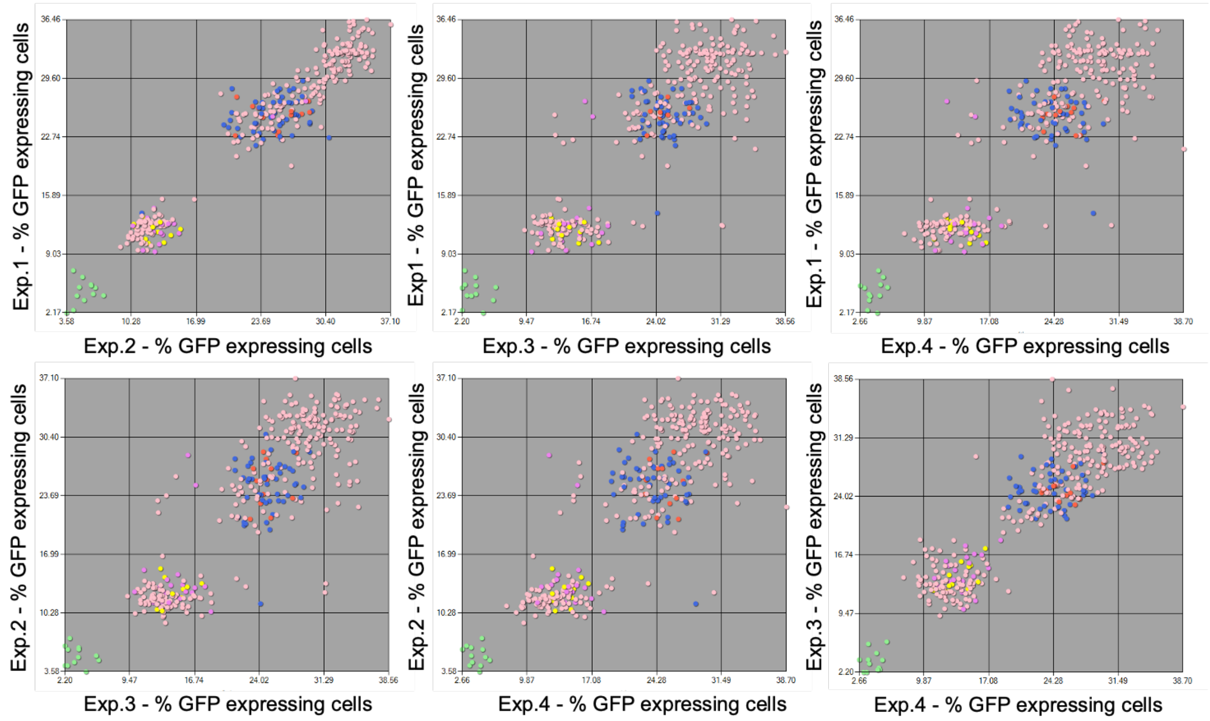

Supplementary figure S3: Dot-plot analysis of counter-screening data coming from four independent experiments. A subset of 57 hit genes promoting or inhibiting *HBD2* expression were selected for validation. Percentage of GFP expressing cells from experiments 1-4 (Exp. 1-4) is reported on X and Y axis. Group data and whole data correlation are presented. Robustness analysis Z' Factor ranges from 0.418 to 0.596. Blue dots: cells treated with the transfection reagent and challenged with *E. coli*; red dots: cells transfected with scramble siRNA and challenged with *E. coli*; yellow dots: cells transfected with scramble siRNA and non-challenged; dark pink dots: non-transfected and non-challenged cells; green dots: cells transfected with GFP siRNA and challenged with *E. coli*; light pink dots: cells transfected with selected siRNA and challenged with *E. coli*. Data analysis of hit genes promoting or inhibiting *HBD2* expression are presented in Fig. 4a and Fig. 4b, respectively.

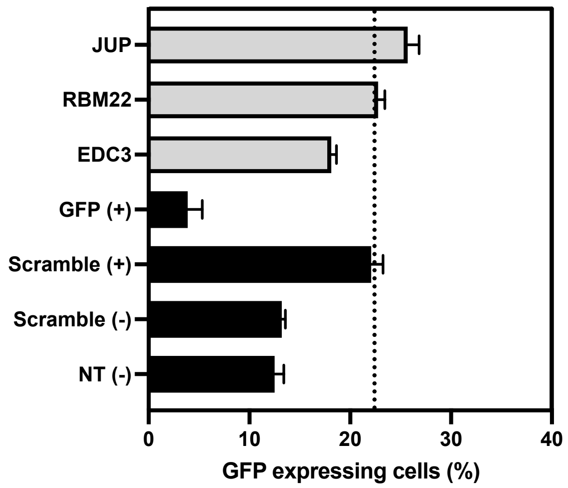

Supplementary figure S4: Hit genes identified by the primary screening but non-validated by the counter-screening. A subset of genes selected among those identified in the primary screening, as promoting or inhibiting *HBD2* expression, was subjected to a validation step by re-analyzing their impact on *HBD2* transcription in 4 independent siRNA transfection experiments. Results are presented as the percentage of GFP expressing cells, as the mean ± s.d. (n = 4 biological replicates). Grey bars: target siRNA; black bars: control siRNA. GFP (+): cells transfected with GFP siRNA and challenged with *E. coli*; scramble (+): cells transfected with scramble siRNA and challenged with *E. coli*; scramble (-): cells transfected with scramble siRNA and non-challenged; NT (-): non-transfected and non-challenged cells. $P > 0.05$ evaluated by One-way Anova (target siRNA vs. scramble (+)).
